# On Variant Discovery in Genomes of Fungal Plant Pathogens

**DOI:** 10.3389/fmicb.2020.00626

**Published:** 2020-04-16

**Authors:** Lizel Potgieter, Alice Feurtey, Julien Y. Dutheil, Eva H. Stukenbrock

**Affiliations:** ^1^Environmental Genomics, Max Planck Institute for Evolutionary Biology, Plön, Germany; ^2^Environmental Genomics, Christian-Albrechts University of Kiel, Kiel, Germany; ^3^Molecular Systems Evolution, Max Planck Institute for Evolutionary Biology, Plön, Germany

**Keywords:** population genomics, fungal pathogens, next-generation sequencing, genome alignment, variant calling, genome assembly

## Abstract

Comparative genome analyses of eukaryotic pathogens including fungi and oomycetes have revealed extensive variability in genome composition and structure. The genomes of individuals from the same population can exhibit different numbers of chromosomes and different organization of chromosomal segments, defining so-called accessory compartments that have been shown to be crucial to pathogenicity in plant-infecting fungi. This high level of structural variation confers a methodological challenge for population genomic analyses. Variant discovery from population sequencing data is typically achieved using established pipelines based on the mapping of short reads to a reference genome. These pipelines have been developed, and extensively used, for eukaryote genomes of both plants and animals, to retrieve single nucleotide polymorphisms and short insertions and deletions. However, they do not permit the inference of large-scale genomic structural variation, as this task typically requires the alignment of complete genome sequences. Here, we compare traditional variant discovery approaches to a pipeline based on *de novo* genome assembly of short read data followed by whole genome alignment, using simulated data sets with properties mimicking that of fungal pathogen genomes. We show that the latter approach exhibits levels of performance comparable to that of read-mapping based methodologies, when used on sequence data with sufficient coverage. We argue that this approach further allows additional types of genomic diversity to be explored, in particular as long-read third-generation sequencing technologies are becoming increasingly available to generate population genomic data.

## Introduction

Comparative genome studies of fungal and oomycete pathogens have revealed highly variable genome architecture and content [reviewed by Raffaele and Kamoun, 2012; Möller and Stukenbrock, 2017]. The genome size and ploidy level of pathogenic fungi and oomycetes can vary significantly between individuals of the same species. Differences can be attributed to the dynamics of transposable elements, chromosome instability, and genome compartmentalization (Möller and Stukenbrock, 2017). Fungal genomes are known to contain accessory compartments that are thought to be relevant for rapid evolution of phytopathogens [reviewed by Croll and McDonald, 2012; Möller and Stukenbrock, 2017]. Typically, these compartments contain a lower density of genes than the core genome, and have a higher content of repetitive elements (Coleman et al., 2009; Ma et al., 2010). Rapidly evolving genome compartments were shown, in some species, to encode virulence determinants (e.g. Does et al., 2016). However, in spite of their functional importance, it is challenging to analyze genetic variation in these regions due to the high extent of structural variability of the genomic sequences.

Population genomic datasets based on next generation sequencing (NGS) can be used to recover genomic variants such as single nucleotide polymorphisms (SNPs), insertions and deletions (indels), and structural variants (SVs). The latter category includes translocations, inversions, duplications, either tandem or interspersed, deletions, and novel sequence insertions (Alkan et al., 2011). Two different frameworks are traditionally used for the detection of variants (Mahmoud et al., 2019). Firstly, a reference-based approach, whereby short read data generated from NGS is mapped on a reference genome, is used to recover SNPs and short indel variants (Horner et al., 2010; El-Metwally et al., 2013). Secondly, from whole genome alignments based on *de novo* assembled genomes. The recovery of small structural variants from short read mapping makes use of mapping distance and orientation information of the reads, as well as read depth and pair-end discordance (Chen et al., 2009; Rausch et al., 2012; Layer et al., 2014). State-of-the-art methods further use a local assembly of the identified inserted material (e.g. (McKenna et al., 2010; Rimmer et al., 2014). Conversely, recovery of large-scale structural variants is typically achieved by first assembling individual genomes, which are then combined into a whole genome alignment (WGA) (Tian et al., 2018). The WGA enables the accurate location of large indels (typically larger than 3 kb) (Nattestad and Schatz, 2016; Tian et al., 2018).

**FIGURE 1:**
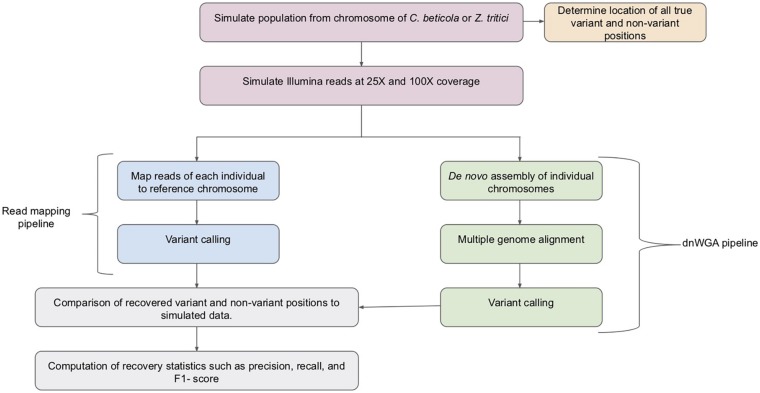
Overview of the pipelines used for the compared approaches. Samples of 20 individuals were simulated from *C. beticola* and *Z. tritici* chromosomes. The simulated chromosome samples were used to establish a set of true variant and non-variant positions. Reads were simulated from the simulated chromosomes at both low (25X) and high (100X) read depth. The reads were then processed by the read mapping pipeline and the dnWGA pipeline. The variant and non-variant positions recovered by each approach at each read depth were then compared to the known introduced variants, and the recovery statistics computed.

While typically used to compare distinct species, if applied at the population level, WGAs potentially provide a crucial resource to conduct population genomic analyses in species with a significant proportion of structural variation since they can, in principle, capture both large and small variants (Faino et al., 2016). However, methods for calling variants in populations from WGAs are currently limited and the available approaches have not been benchmarked with fungal genome data. In this study, we take the first step to compare variant discovery approaches for population genomic analyses of fungal pathogen genomes. We assess the accuracy of a pipeline based on *de novo* genome assembly followed by whole genome alignment (referred to as dnWGA, Figure 1) to simultaneously recover single nucleotide polymorphisms (SNPs) and large structural variants.

Comparisons of methods for genome assembly and variant calling have so far been performed on human data or simulated data sets with human-like properties (Hwang et al., 2015; Wu et al., 2017; Bian et al., 2018). Several studies reported that the performance of SNP callers depends on the sequence complexity, coverage, and read filtering criteria used (Hwang et al., 2015; Sandmann et al., 2017). In particular, most variant calling pipelines have trouble accurately determining variants in repeat rich regions (Krusche et al., 2019). Reference based variant calling and dnWGA approaches can be compared using simulated data sets, for which the “true” set of variants is known (Wu et al., 2017). The resulting called positions can be subsequently classified into one of four categories: (1) correctly identified variant positions [true positives (TP)], (2) correctly identified non-variant positions [true negatives (TN)], (3) variants incorrectly called in non-variant positions [false positives (FP)], and (4) variable positions that were not identified by the calling method [false negatives (FN)] (Rosner, 2006). The proportion of variants falling in each of these categories allows to compute several measures of performance (Goutte and Gaussier, 2005). Hereby “precision” is defined as the proportions of correctly inferred positives (TP/(TP + FP)), while the “recall” measure denotes the proportion of variable positions that were recovered (TP/(TP + FN)). Like many classification procedures, most variant calling methods are subject to a trade-off between precision (the higher the precision value, the more confident we can be in the prediction), and recall (the higher the recall value, the more exhaustive the variant discovery is). The performance of a given method along this trade-off can be captured by the F1 score, defined as the harmonic mean of the precision and recall values:

(1) F1=2*(recall*precision)/(recall+precision)

The F1 score is, therefore, a global measure of the reliability of the variant discovery method (Goutte and Gaussier, 2005).

Several studies have demonstrated that the data used for benchmarking of variant callers is critical (e.g. Hwang et al., 2015; Sandmann et al., 2017; Wu et al., 2017; Bian et al., 2018). Notably, human population genomic data have been considered producing well-defined benchmarking tools, including the “Genome in a Bottle” project that has published a set of high-confidence variants for a reference genome (see^1^ : Hwang et al., 2015). Since fungal pathogen genomes differ from human genomes in many aspects, we here aimed to compare variant calling approaches on data sets specifically mimicking the characteristics of fungal pathogen genomes, including accessory genome compartments and high nucleotide diversity.

## Method Overview

To compare the performance of dnWGA and reference-based mapping for variant calling, we generated population genomic data sets from chromosomal sequences of two fungal plant pathogens, *Cercospora beticola* and *Zymoseptoria tritici* using simulations (see Supplementary Methods for a detailed description of methods and materials). We selected these two different species with distinct repeat content, since repeats are known to hamper the variant calling process. While the *C. beticola* chromosome was virtually deprived of repeats (0.2% of 5.8 Mb) (de Jonge et al., 2018), a comparatively high proportion [11% of 6.2 Mb (Grandaubert et al., 2015)] is annotated in the chromosome of *Z. tritici*. We employed the chromosomes to simulate a population genomic data set that resembled empirical population genomic data. The genetic diversity of the simulated populations, measured by Watterson’s theta, was 0.0077 and 0.0073 for *C. beticola* and *Z. tritici*, respectively (Watterson, 1975). The simulated population data sets comprised SNPs, indels, and accessory genome segments at known positions allowing us to evaluate the variant recovery (Figure 1).

We simulated NGS reads from the simulated genomes with both low (25X) and high (100X) sequencing coverage. To compare the efficacy of SNP discovery methods on each of the four data sets (two species, and two depths of coverage), we computed the recall, precision, and F1 scores in each case. We further assess whether the large SV were properly recovered by the dnWGAs. Details on the data generation and analyses are provided in the Supplementary Text.

## Results and Discussion

**FIGURE 2:**
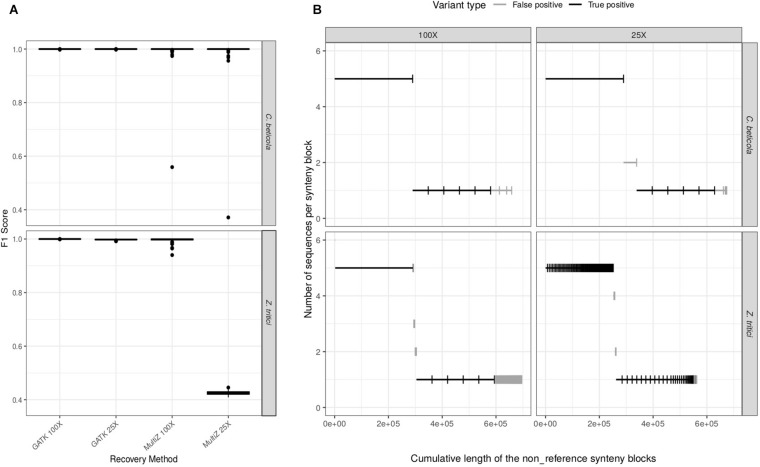
Comparison of the efficacy of variant calling pipelines. **(A)** distribution of the F1 scores of SNP recovery methods for *C. beticola* and *Z. tritici* at 25X and 100X sequencing depths. **(B)** Recovery of non-reference sequences (accessory regions) in the whole-genome alignments (WGA) for both species and at both sequencing depths. Six accessory regions have been introduced in the simulated chromosomes: one long region (290 kb) in five individuals, and five smaller regions (58 kb) in one individual. Each segment corresponds to a synteny block in the WGA. Segments have been ordered by decreasing frequency in the sample (*y*-axis). The *x*-axis represents the resulting cumulative sum of segment lengths. Each segment is classified as true (black) or false (gray) positive, according to whether it corresponds to a simulated insertion or not. A false positive is a sequence detected as non-reference in the final WGA but which was not inserted during the simulation process (i.e. an artifact of either assembly or alignment) or a sequence inserted during the simulations but recovered in only some of the genomes and/or at an incorrect position.

While WGAs are used to infer structural variation, how well they can recover single-nucleotide variation has not been systematically tested in fungi. We first set out to compare the performance of SNP recovery of dnWGA and reference-based approaches. We specifically ask how the sequencing depth and repeat content of the genomes affect the relative performance of the two methods: the F1 score of the reference-based approach was found to be higher than 99.7% for both *Z. tritici* and *C. beticola*, at low (25X) and high (100X) coverage. The F1 score of the dnWGA approach, however, depends on the sequence depth and the species (Figure 2A). When using high coverage data, the F1 score in both species reaches 99.9%. When low coverage sequencing was used, however, the F1 score was found to be similarly high (99.9%) for *C. beticola*, but only 43% for the *Z. tritici* data set. This effect is essentially due to the precision getting as low as 28%, while the recall value remains comparatively high (93%), suggesting that the false positive rate is high for the repeat rich chromosome with low sequencing coverage (Supplementary Table S1).

We further investigated the drop of performance at low coverage of the dnWGA approach in the repeat-rich *Z. tritici* data set by comparing the genome assemblies. N50 was equal to 219 kb with the 100X data set, but only 12 kb when using a 25X read depth (Supplementary Tables S2, S3). In comparison, the *de novo* assemblies of the *C. beticola* chromosomes showed a comparable N50 of 1.8 Mb and 1.7 Mb at 100X and 25X, respectively (Supplementary Tables S4, S5) underlining the impact of high repeat content in *Z. tritici* on chromosome assemblies. We used Quast (Gurevich et al., 2013) to further quantify the accuracy of the assembled genomes and identify misassemblies, defined as regions of the *de novo* assemblies that did not align to the original chromosome at the correct positions. In the repeat poor *C. beticola* data set, the number of misassemblies remained comparable for both sequencing depths. For the *Z. tritici* data set, however, we find four times more misassemblies in the 25X than in the 100X data. Therefore, we conclude that the low performance of the dnWGA procedure at low sequencing coverage is essentially due to failure of *de novo* assembling the chromosome sequences in the presence of a higher repeat content.

We then investigated whether the dnWGA approach could recover the simulated accessory regions. Such regions should appear in the WGA as synteny blocks that do not contain the reference sequence. We extracted such synteny blocks from the WGA and compared their size and sequence to the known introduced regions to identify false and true positives. In the *C. beticola* alignment, the accessory regions were recovered entirely as single regions and in all the chromosomes they were introduced into (Figure 2B). The accessory regions introduced in the *Z. tritici* chromosomes could be recovered with a similar level of quality at a depth of 100X. Conversely, at 25X, all recovered insertions were fragmented, but 542 out of the 580 kb inserted (93%) were recovered (Figure 2B). False positive regions (non-reference DNA fragments that did not match with the introduced sequences in all WGA) were also detected in all data sets, with a total size ranging from 15 kb to 116 kb per WGA. These regions were found to be comparatively small, and more abundant in the repeat-rich *Z. tritici* data set. In summary, we find that dnWGA allows the recovery of accessory regions in population genomic datasets. For genomes with a low frequency of repeats high performance is achieved even with low coverage data, while high coverage data is required in the presence of repeats.

## Perspective

The genomes of eukaryote pathogens including fungi and oomycetes can comprise extensive structural variation such as accessory regions, not found in reference genomes. So far, methods to analyze genetic variation in populations of individuals with different genome content and structure are sparse. Whole genome alignment of *de novo* assembled genomes permits the joint analysis of genetic variation ranging from single nucleotide substitutions to large structural variation. We here show that SNPs can be called from WGAs with a precision similar to that of mapping-based approaches when sufficient sequencing coverage is achieved. We note that with our benchmark based on fungal data, the performance of the dnWGA approach was higher than what was observed in previous comparisons performed on human datasets, where the precision and recall were 87 and 50% at 20X, and 93 and 56% at 50X (Wu et al., 2017). Moreover, the dnWGA approach also allowed us to recover accessory chromosome fragments, genomic features that were shown to occur frequently in fungal genomes. The computational framework based on *de novo* assembled genomes, therefore, also potentially allows for the analyses of genome segments encoding orphan genes, the comparison of highly dynamic genome compartments, the detection of accessory chromosomes, and the study of repeat dynamics within a population.

In genomes with high frequencies of SVs and accessory regions, the use of dnWGA allows for reference-based mapping to be skipped entirely for variant discovery. However, current methods based on WGA are computationally more demanding than reference-based mapping approaches. As assembly algorithms are improving in quality and efficiency, fostered by the development of long-read sequencing technologies, whole genome alignment constitutes the next methodological challenge. Current state-of-the-art methods are designed for interspecific comparisons and relatively small sample sizes (typically less than 100 genomes). As such, they are not sized to cope with population genomic data sets, for which mechanisms such as recombination can no longer be ignored and prohibits the use of a single guide tree when aligning multiple genomes. The average higher similarity of genomes from a single species, however, should permit more efficient alignment algorithms. A new generation of genome aligners is, therefore, needed to exploit the full potential of long-read sequencing technologies to characterize genome variation in populations.

## Data Availability Statement

Raw data and scripts necessary to reproduce the analyses are available at https://gitlab.gwdg.de/alice.feurtey/variant-discovery-methods and doi: 10.5281/zenodo.3696563.

## Author Contributions

LP and AF carried out the implementation of the framework with input from JD. LP analyzed the data and wrote the manuscript with input from all authors. JD and ES conceived the study and were in charge of overall direction and planning.

## Conflict of Interest

The authors declare that the research was conducted in the absence of any commercial or financial relationships that could be construed as a potential conflict of interest.

## Footnotes

1 https://www.nist.gov/programs-projects/genome-bottle
