# Supplementary material for: On Variant Discovery in Genomes of Fungal Plant Pathogens
Source: Front Microbiol. 2020 Apr 16;11:626. doi: 10.3389/fmicb.2020.00626 (PMC7176817; doi:10.3389/fmicb.2020.00626)
Supplement: Supplementary file 1 [file Data_Sheet_1.docx]

On variant discovery in genomes of fungal plant pathogens

Lizel Potgieter^1,2^*, Alice Feurtey^1^, Julien Y. Dutheil^3^, Eva H. Stukenbrock^1,2^

# Supplementary: Materials and Methods

All custom scripts cited in this text are available at <https://gitlab.gwdg.de/alice.feurtey/variant-discovery-methods>

All data used in this text are available at the DOI: 10.5281/zenodo.3696563

# Population Simulation

In order to simulate data with different characteristics (such as sequence composition or repeat content) similar to that of fungal pathogens, we based our simulations on two Dothideomycete species for which a complete genome sequence was available: *Cercospora beticola,* the causative agent of cercospora leaf spot on sugar beet as well as a variety of uncultivated plants (de Jonge et al., 2018), and *Zymoseptoria tritici*, the causal agent of the wheat disease septoria leaf blotch (Goodwin et al., 2011).

The first simulations were based on chromosome 1 of the *C. beticola* (accession number: PRJNA270309, chromosome: CM008499.1). The chromosome sequence was extracted and all ambiguous characters were removed. The final sequence used here includes 5.8 Mb and had an average GC content of 50.3%. The second simulations were based on the first chromosome of *Z. tritici* (accession number: PRJNA19047, chromosome: CM001196.1). The sequence used was 6.1 Mb in length and had an average GC content of 53.06%. The repeat content of these chromosomes is different: 0.2% for *C. beticola*, and 11% in *Z. tritici*, allowing us to take into account the impact of repeats on the variant recovery.

From the two chromosome sequences, we simulated a population of 20 individuals with ALF (Dalquen et al., 2012). ALF allows simulating full genome sequence from an ancestral sequence under a given evolutionary model along a tree. We made use of the native birth-death model from ALF to generate a random tree, and applied an HKY substitution model (Hasegawa et al., 1985). A total tree height of 5 and 0.3 were set for the *C. beticola* and *Z. tritici* simulations respectively, to ensure that resulting samples have a Watterson’s theta value of 10^-3^ to resemble empirical data sets (Stukenbrock and Dutheil, 2018).

We used MafFilter to determine the variant positions from the multiple sequence alignment produced by ALF (Dutheil et al., 2014). We used simuG (Yue and Liti, 2018) to simulate indels according to the genealogies simulated by ALF and a custom python script to insert them into the simulated genomes. In one branch of the tree an accessory region that was 5% of the total genome size was inserted, and 5 orphan regions each 1% the size of the total chromosome were added in a single individual. From these simulated genomes Illumina reads of 25X and 100X coverage were generated with ART (Huang et al., 2012). The simulated genomic reads were used in two separate pipelines (Figure 1).

# Short Variant Calling

In the following, we define the reference chromosome as the ancestral sequence from which the synthetic population was simulated. In the reference-based mapping pipeline, the short reads were mapped to the reference chromosome using BWA-mem (Li and Durbin, 2009). The alignment files were sorted, indexed, and duplicates were removed with the SAMtools (Li et al., 2009), and further processed with the Picard Tools (http://broadinstitute.github.io/picard/). Short variant discovery was done with GATK v4.1.0.0 (Auwera et al., 2013; DePristo et al., 2011; McKenna et al., 2010). HaplotypeCaller was used to produce gVCF files. These were combined with CombineGVCFs, and the resulting SNP coordinates and frequencies were written in a VCF file with GenotypeGVCFs, following the GATK Best Practices Workflow (<https://gatk.broadinstitute.org/hc/en-us/articles/360035535932-Germline-short-variant-discovery-SNPs-Indels->).

In the dnWGA pipeline, the short reads were *de novo* assembled with SPAdes (Bankevich et al., 2012). The quality of each assembly was determined by QUAST (Gurevich et al., 2013). Whole genome alignment of the *de novo* assembled genomes with the reference chromosome by MultiZ (Blanchette et al., 2004) was run in parallel with GNU parallel (Tange, 2011). The alignment was projected on the reference sequence with maf_project (Blanchette et al., 2004), and SNPs were called using MafFilter (Dutheil et al., 2014).

The variants called by the two pipelines were stored as VCF files and filtered to remove all missing data and indels with the VCFtools (Danecek et al., 2011). We compared the filtered variants using a custom R script. We computed the precision, recall, and F1 score of each method (Goutte and Gaussier, 2005) by comparing the variants discovered by each pipeline to the true set of variants from the simulations.

# Recovery of Accessory Regions

The multiple genome alignments from MultiZ were used to call accessory regions, which are not part of the reference genome. The alignments were filtered with MafFilter to remove all blocks smaller than 1 kb. A custom python script was used to determine the identity of scaffolds contained in alignment blocks. Another custom R script was used to extract the coordinates of the synteny blocks that did not contain any reference sequence. The corresponding regions were extracted from the assemblies with the bedtools getfasta function (Quinlan and Hall, 2010). These regions were then compared to the simulated genomes with BLASTn (Altschul et al., 1990) to determine the location of the unaligned regions. These were compared to the known locations of the inserted accessory regions in order to identify true and false positives in the recovery of accessory regions, with a true positive being defined as a block in which the correct number of sequences are recovered and matching the correct coordinates.

# References

Altschul, S.F., Gish, W., Miller, W., Myers, E.W., and Lipman, D.J. (1990). Basic local alignment search tool. J. Mol. Biol. *215*, 403–410.

Auwera, G.A.V. der, Carneiro, M.O., Hartl, C., Poplin, R., Angel, G. del, Levy‐Moonshine, A., Jordan, T., Shakir, K., Roazen, D., Thibault, J., et al. (2013). From FastQ Data to High-Confidence Variant Calls: The Genome Analysis Toolkit Best Practices Pipeline. Curr. Protoc. Bioinforma. *43*, 11.10.1-11.10.33.

Bankevich, A., Nurk, S., Antipov, D., Gurevich, A.A., Dvorkin, M., Kulikov, A.S., Lesin, V.M., Nikolenko, S.I., Pham, S., Prjibelski, A.D., et al. (2012). SPAdes: A New Genome Assembly Algorithm and Its Applications to Single-Cell Sequencing. J. Comput. Biol. *19*, 455–477.

Blanchette, M., Kent, W.J., Riemer, C., Elnitski, L., Smit, A.F.A., Roskin, K.M., Baertsch, R., Rosenbloom, K., Clawson, H., Green, E.D., et al. (2004). Aligning Multiple Genomic Sequences With the Threaded Blockset Aligner. Genome Res. *14*, 708–715.

Dalquen, D.A., Anisimova, M., Gonnet, G.H., and Dessimoz, C. (2012). ALF—A Simulation Framework for Genome Evolution. Mol. Biol. Evol. *29*, 1115–1123.

Danecek, P., Auton, A., Abecasis, G., Albers, C.A., Banks, E., DePristo, M.A., Handsaker, R.E., Lunter, G., Marth, G.T., Sherry, S.T., et al. (2011). The variant call format and VCFtools. Bioinformatics *27*, 2156–2158.

DePristo, M.A., Banks, E., Poplin, R., Garimella, K.V., Maguire, J.R., Hartl, C., Philippakis, A.A., del Angel, G., Rivas, M.A., Hanna, M., et al. (2011). A framework for variation discovery and genotyping using next-generation DNA sequencing data. Nat. Genet. *43*, 491–498.

Dutheil, J.Y., Gaillard, S., and Stukenbrock, E.H. (2014). MafFilter: a highly flexible and extensible multiple genome alignment files processor. BMC Genomics *15*, 53.

Goodwin, S.B., M’Barek, S.B., Dhillon, B., Wittenberg, A.H.J., Crane, C.F., Hane, J.K., Foster, A.J., Lee, T.A.J.V. der, Grimwood, J., Aerts, A., et al. (2011). Finished Genome of the Fungal Wheat Pathogen *Mycosphaerella graminicola* Reveals Dispensome Structure, Chromosome Plasticity, and Stealth Pathogenesis. PLOS Genet. *7*, e1002070.

Goutte, C., and Gaussier, E. (2005). A Probabilistic Interpretation of Precision, Recall and F-Score, with Implication for Evaluation. In Advances in Information Retrieval, D.E. Losada, and J.M. Fernández-Luna, eds. (Springer Berlin Heidelberg), pp. 345–359.

Gurevich, A., Saveliev, V., Vyahhi, N., and Tesler, G. (2013). QUAST: quality assessment tool for genome assemblies. Bioinformatics *29*, 1072–1075.

Hasegawa, M., Kishino, H., and Yano, T. (1985). Dating of the human-ape splitting by a molecular clock of mitochondrial DNA. J. Mol. Evol. *22*, 160–174.

Huang, W., Li, L., Myers, J.R., and Marth, G.T. (2012). ART: a next-generation sequencing read simulator. Bioinformatics *28*, 593–594.

de Jonge, R., Ebert, M.K., Huitt-Roehl, C.R., Pal, P., Suttle, J.C., Spanner, R.E., Neubauer, J.D., Jurick, W.M., Stott, K.A., Secor, G.A., et al. (2018). Gene cluster conservation provides insight into cercosporin biosynthesis and extends production to the genus Colletotrichum. Proc. Natl. Acad. Sci. 201712798.

Li, H., and Durbin, R. (2009). Fast and accurate short read alignment with Burrows-Wheeler transform. Bioinforma. Oxf. Engl. *25*, 1754–1760.

Li, H., Handsaker, B., Wysoker, A., Fennell, T., Ruan, J., Homer, N., Marth, G., Abecasis, G., Durbin, R., and 1000 Genome Project Data Processing Subgroup (2009). The Sequence Alignment/Map format and SAMtools. Bioinforma. Oxf. Engl. *25*, 2078–2079.

McKenna, A., Hanna, M., Banks, E., Sivachenko, A., Cibulskis, K., Kernytsky, A., Garimella, K., Altshuler, D., Gabriel, S., Daly, M., et al. (2010). The Genome Analysis Toolkit: A MapReduce framework for analyzing next-generation DNA sequencing data. Genome Res. *20*, 1297–1303.

Quinlan, A.R., and Hall, I.M. (2010). BEDTools: a flexible suite of utilities for comparing genomic features. Bioinformatics *26*, 841–842.

Stukenbrock, E.H., and Dutheil, J.Y. (2018). Fine-Scale Recombination Maps of Fungal Plant Pathogens Reveal Dynamic Recombination Landscapes and Intragenic Hotspots. Genetics *208*, 1209–1229.

Tange, O. (2011). Gnu parallel-the command-line power tool.; login: The USENIX Magazine, 36 (1): 42–47 (Feb).

Yue, J.-X., and Liti, G. (2018). simuG: a general-purpose genome simulator. BioRxiv 491498.
